# Association of health asset value with subjective well-being, depression, health management strategy and habits in South Korea

**DOI:** 10.1038/s41598-022-23099-8

**Published:** 2022-10-27

**Authors:** Young Ho Yun, Hyejeong Yoon, Eunwoo Park

**Affiliations:** 1 Department of Human Systems Medicine, Seoul National University College of Medicine, Seoul, Korea; 2 Department of Family Medicine, Seoul National University College of Medicine, 103 Daehak-ro, Jongno-gu, Seoul, 110-799 Korea; 3 Seoul National University College of Liberal Studies, Seoul, Korea; 4 Seoul National University School of Law, Seoul, Korea

**Keywords:** Health care, Signs and symptoms

## Abstract

This study aims to measure the monetary value of health asset based on the self-reported health status and rate of health asset value (HAV), and to evaluate its application to the subjective well-being and health competency of a representative sample of South Korea. From March to April 2021, 1000 participants were randomly sampled nationwide in South Korea and administered questionnaires including self-reported rate of health asset value and health status, the Subjective Well-Being Index (SWBI), Patient Health Questionnaire-9 (PHQ-9), Smart Management Strategy for Health Assessment Tool (SAT), and 11 health habits. In multiple stepwise logistic regression model adjusted for basic demographic variables (age, sex, region, monthly income level, and comorbidity), current HAV was independently associated positively with SWBI (adjusted odds ratio [aOR], 4.32; confidence interval [CI] 2.27–8.23) and negatively with PHQ-9 (aOR 0.68; 95% CI 0.51–0.90). Core (aOR 1.66; CI 1.25–2.19), Preparation (aOR 1.79; CI 1.24–2.59), and Implementation Strategy scores of SAT (aOR 1.79; CI 1.26–2.55) were independently associated positively with current HAV. All 11 health habits were independently associated positively with current HAV (aOR range from 1.80 to 3.19). The HAV approach offers a new monetary value of health that can be used in making individual or political decisions of improving health or reducing health inequity.

## Introduction

Promoting health and tackling inequalities are global issues^1,2^ and the efforts to improve health and reduce health inequalities in individual and social dimensions should be a priority area for future public health^3^.

Recently, there has been increased interest in ‘health asset’ approaches that create positive health value^1,4^. Good health allows people to participate in the workplace and social life, create human and social value, and has a significant influence on human beings. Health can be viewed as assets worthy of investment for individuals and society to prosper^2^. The valuation of health asset could provide additional insight into the opportunity value of health for individual or social decision-making in promoting health and tackling health inequalities^5^.

General Health Status (GHS), that is, subjective health status was reported and compared across Organization for Economic Cooperation and Development (OECD) countries. Economic performance may be affected by the general health of the population, and gross domestic product (GDP) growth might be associated with the self-reported health status of a country^2,6^. Therefore, health assets with self-reported health status, such as GHS, can be evaluated. However, there is a pressing need to incorporate multidimensional health based on the World Health Organization’s (WHO) definition to help reveal both individual and population-level health asset value (HAV).

For individuals and communities, the health asset approach includes the skills and motivation to take better control of their own health and work more collaboratively with public health services to achieve their health vision^1^.

However, asset-based approaches have failed to show benefit for mental health, well-being, and health management in individuals, communities, and corporate.

As there is growing recognition of the need beyond the current approach in health economics^7^, we have estimated value of health assets and the asset cost of poor health by using household net income and health status as a function of individual health status to capture the cumulative effect of the financial consequences of poor health^5^. Research on the relationship between the HAV, quality of life, copying strategies and habit is needed to assist individuals and societies in the decision-making process to promote health and address health inequalities.

Although societal goals still generally focus on economic growth, income, and employment in South Korea, good health allows people to participate in family life, the workplace, and the community, and significantly influences overall well-being. Health can be perceived as human capital and, thus, as an input to other necessary social values that allow people in South Korea. Therefore, for individuals and society to grow, individuals might tend to value health more highly than their incomes or careers^1,2^ and health should be viewed as an asset worth investing in^2^.

This study aims to measure monetary value of health asset based on self-reported health status and HAV, and to evaluate its application to the subjective well-being, depression, health management strategy, and habit of a representative sample of South Korea. We hypothesized that higher HAV would predict low depression, better well-being, and health management competency. Gaining insight into self-rated health asset valuation can improve individuals and society by guiding the decision-making process in promoting the health and tackling health inequalities.

## Methods

### Participants and procedures

This is cross-sctional study. The data for this study were collected from March 2 to April 9, 2021, from a sample of the general Korean population. The inclusion criteria were as follows: (1) people aged ≥ 20 years, (2) people able to understand the objectives of the survey. The exclusion criteria were as follows: (1) people who could not speak, hear, or read Korean, (2) those had difficulty in understanding the contents of the questionnaire due to vision or hearing problems. Participants ranged in age from their 20 to 70 s, and their places of residence spanned 17 major cities and local districts in South Korea. We recruited participants within age and sex strata by region, and we used the probability proportional to size technique to select a representative national sample in accordance with the 2019 Korean census, particularly when the sizes of the sample groups differed^8^. We randomly selected 1000 people, which is an appropriate sample size for reliable parameter estimation. Of the 1800 eligible participants, 1000 responded (55.6% response rate) to the self-reported questionnaire in the presence of trained research assistant interviewers, who received informed consent from participants, answered questions and provided further explanations of the study. K Stats Co., Ltd. (Seoul, Korea) conducted the survey. Ethics approval for this study was obtained from the Institutional Review Board of Seoul National University as an IRB review exempt study (SNUH; IRB No.2102-098-1197).

### Measurement

To measure the influences of different aspects of self-rated health on health assets and their valuations, we asked respondents to assess themselves in terms of four types of health. The relevant questions are as follows: ‘Physical health is the state of normal physical strength, without any disease or injury. What do you think about your physical health status?’ ‘Mental health is the state of being mentally stable and being able to overcome stress. What do you think about your mental health status?’ ‘Social health is the state of having good social relationships and carrying out one’s work properly. What do you think about your social health status?’ ‘Spiritual health is the state of adding meaning to life through volunteering, religious experiences, and meditation. What do you think about your spiritual health status?’ These items were rated on a five-point Likert scale, with 1, 2, 3, 4, and 5 corresponding to ‘*Excellent*’, ‘*Very good*, ‘*Good*’, ‘*Poor*’, and ‘*Very poor*’^9^. Weighing scores of four health aspects (physical, mental, social, spiritual) were based on multiple regression with partial R^2^, which the sum of weighing values of four health is 1.

There is no ‘golden standard’ for health asset valuation. We calculated HAV with self-reported health status, health asset value rate (HAVR), weighting score of four health aspects and annual income into a single metric. Our HAVR was based on the contingent valuation, which is the most common type of stated preference methods that used to value non-market goods^10^. HAVR asked respondents to value the loss of health asset with a percentage of annual income loss using a hypothetical scenario of a 10% loss of health asset, and indirectly extracts their valuation of health asset: ‘If you lose 10% of your health assets for each health type (physical, mental, social, spiritual) in 1 year, what percentage of your annual income would you think you lost?’.

The participants completed the questionnaire including Subjective Well-Being Index (SWBI)^11^ for subjective well-being and Patient Health Questionnaire-9 (PHQ-9)^12^ for depression. The participants were asked to measure their self-management strategies of health with smart management strategy for health assessment tool (SAT), which comprises three sets and 16 factors related to health strategies encompassing core strategies, preparation strategies, and implementation strategies^13^. The validity and reliability of all three questionnaires have been verified.

Additionally, participants reported 11 health habits^9^ with five scales (pre-contemplation, contemplation, preparation, action, and maintenance) based on the Transtheoretical Model^14^. We also collected information on sex, age, monthly income, and comorbidity for each respondent.

### Statistical analysis

We used the G-power program to set the appropriate sample size for the effect size as (0.2), α (0.05), and 1 − β (0.95). Considering gender, age (20’s, 30’s, 40’s, 50’s, 60’s, or older), and regional size (special district, metropolitan area, city, county), the number of groups with the appropriate sample size was 1000.

Multiple logistic regression analyses were conducted to: (1) examine whether socioeconomic characteristics are independently associated with HAV; (2) assess the association of HAV with subjective well-being and depression, adjusted for age, sex, income, and comorbidity; and (3) identify the association of SAT and health habits with HAV adjusted for age, sex, income, and comorbidity. The problematic group of SAT was defined as one with each SAT set score of 66.6 or less^13^. Only those who answered ‘maintenance’ for health habits were considered the maintenance group.

In all analyses, the adjusted odds ratios were reported (aORs) with 95% confidence intervals (CI) as the results, and statistical significance was defined as a two-sided *P* value less than 0.05. The final multivariate analysis results are considered meaningful. We used SAS statistical software (version 9.4; SAS Institute, Cary, North Carolina, USA) for all analyses.

### Ethics approval and consent to participate

All procedures performed in this study involving human participants were in accordance with the ethical standards of the Institutional Research Committee and with the 1964 Helsinki Declaration and its later amendments or comparable ethical standards. Our study on validation of the SAT-Parentship was reviewed and approved by the Institutional Review Board (IRB) of the Seoul National University Hospital [C-2001-164-1098]. Informed consent was obtained from all individual participants included in the study.

### Informed consent

Participants who gave voluntary informed consent were only considered as subjects of the study and allowed to proceed with the study.

## Results

### Baseline characteristics

Most people were married, employed, and resided in urban areas in apartments or houses. (Table 1) The mean age of the study participants is 48, with a standard deviation of 14.65 years.

**Table 1:** Participants’ sociodemographic characteristics.

Variable	Description	N	%
Age	20–29	166	16.60
	30–39	166	16.60
	40–49	205	20.50
	50–59	209	20.90
	60–69	164	16.40
	≥ 70	90	9.00
Sex	Male	503	50.30
	Female	497	49.70
Education	College graduate	541	54.10
	High school graduate	361	36.10
	Middle school or less	90	9.80
Income	≥ 5000	276	27.60
	4000–5000	274	27.50
	3000–4000	228	22.80
	< 3000	221	22.10
Marriage	Married	714	71.40
	Not married	286	28.60
Residence	Urban	460	46.00
	Rural/suburban	540	54.00
Religion	Having religion	360	36.00
	No religion	640	64.00
Job-status	Occupied	747	74.70
	Non-occupied	253	25.30

### Association among basic demographic variables with current HAV, multiple stepwise model

In multiple stepwise logistic regression model with basic demographic variables (age, sex, region, monthly income level, and comorbidity), only monthly income level was independently associated with current HAV (aOR 6.77; 95% CI 4.65–9.84). (Table 2).

**Table 2:** Associations of demographic characteristics with current health asset value in the participants (*n* = 1000), multiple stepwise model.

	Current HAV (median, 610.1) (High vs. low)	
	aOR	95% CI
**Age**		
Young (≤ 50)	1 (Ref)	
Older		
**Sex**		
Male	1 (Ref)	
Female		
**Household monthly income**		
Low	1 (Ref)	
High (300 ≤)	6.77	4.65–9.84
**Comorbidity**		
No	1 (Ref)	
Yes		

*HAV* health asset value, *aOR* adjusted odds ratios, *CI* confidence interval, *Ref* reference.

### Associations of current HAV with subjective well-being and depression, multiple stepwise model

In multiple stepwise logistic regression model adjusted for basic demographic variables (age, sex, region, monthly income level, and comorbidity), current HAV was independently associated positively with SWBI (aOR 4.32; CI 2.27–8.23) and negatively with PHQ-9 (aOR 0.68; 95% CI 0.51–0.90) (Table 3).

**Table3:** Associations of current health asset with subjective well-being and depression, adjusted for age, sex, income, and comorbidity (*n* = 1000), multiple stepwise model*.

	SWBI		PHQ-9	
	aOR	95% CI	aOR	95% CI
**Current HAV (median, 610.1)** **(high vs. low)**				
Low	1 (Ref)		1 (Ref)	
High	4.32	2.27–8.23	0.68	0.51–0.90

*HAV* health asset value, *aOR* adjusted odds ratios, *CI* confidence interval, *Ref* reference, *SWBI* Subjective Well-Being Index, *PHQ-9* Patient Health Questionnaire-9.

*Multiple stepwise logistic regression models selected significant variables with p-value of stay = 0.05, leave = 0.05, adjusted with age (≤ 50 vs. > 50), sex (male vs. female), household monthly income (< 3000$ vs. 3000$ ≤), and comorbidity (no vs. yes).

### Associations of health management strategies with Current HAV, multiple stepwise model

In multiple stepwise logistic regression model adjusted for basic demographic variables (age, sex, region, monthly income level, and comorbidity), Core SAT (aOR 1.66; CI 1.25–2.19), Preparation SAT (aOR 1.79; CI 1.24–2.59), and Implementation SAT (aOR 1.79; CI 1.26–2.55) were independently associated positively with current HAV (Table 4).

**Table 4:** Associations of current health asset value with health management strategies, adjusted for age, sex, income, and comorbidity (*n* = 1000), multiple stepwise model*.

	Core SAT		Preparation SAT		Implementation SAT	
	aOR	95% CI	aOR	95% CI	aOR	95% CI
**Current HAV (median, 610.1)** **(High vs. low)**						
Low	1 (Ref)		1 (Ref)		1 (Ref)	
High	1.66	1.25–2.19	1.79	1.24–2.59	1.79	1.26–2.55

*HAV* health asset value, *aOR* adjusted odds ratios, *CI* confidence interval, *Ref* reference, *SAT* Smart Management Strategy for Health (SMASH) Assessment Tool.

*Multiple stepwise logistic regression models selected significant variables with p-value of stay = 0.05, leave = 0.05, adjusted with age (≤ 50 vs. > 50), sex (male vs. female), household monthly income (< 3000$ vs. 3000$ ≤), and comorbidity (no vs. yes).

### Associations of current HAV with maintenance of 11 health habits with multiple stepwise model

In multiple stepwise logistic regression model with basic demographic variables (age, sex, region, monthly income level, and comorbidity), all 11 health habits were independently associated positively with current HAV (aOR range from 1.80 to 3.19) (Table 5).

**Table 5:** Associations of current health asset value with maintenance of health behaviors for more than 6 months, for age, sex, income, and comorbidity (*n* = 1000), multiple stepwise model*.

	Current HAV (median, 610.1) (High vs. low)	
	aOR	95% CI
**Regular exercise**		
≤ Action	1 (Ref)	
Maintenance	1.85	1.36–2.53
**Balanced diet**		
≤ Action	1 (Ref)	
Maintenance	2.46	1.81–3.35
**Regular check-ups**		
≤ Action	1 (Ref)	
Maintenance	2.30	1.71–3.08
**Smoking cessation**		
≤ Action	1 (Ref)	
Maintenance	2.01	1.42–2.85
**Drinking cessation**		
≤ Action	1 (Ref)	
Maintenance	1.80	1.31–2.47
**Work-life balance**		
≤ Action	1 (Ref)	
Maintenance	2.33	1.67–3.27
**Positive thinking**		
≤ Action	1 (Ref)	
Maintenance	3.04	2.30–4.01
**Proactive living**		
≤ Action	1 (Ref)	
Maintenance	3.19	2.39–4.27
**Living with loved ones**		
≤ Action	1 (Ref)	
Maintenance	3.10	2.32–4.14
**Helping others**		
≤ Action	1 (Ref)	
Maintenance	1.91	1.30–2.83
**Regular religious life**		
≤ Action	1 (Ref)	
Maintenance	2.09	1.41–3.09

*HAV* health asset value, *aOR* adjusted odds ratios, *Ref* reference.

^†^Multiple stepwise logistic regression models selected significant variables with p-value of stay = 0.05, leave = 0.05, adjusted with age (≤ 50 vs. > 50), sex (male vs. female), household monthly income (< 3000$ vs. 3000$ ≤), and comorbidity (no vs. yes).

## Discussion

Our health asset valuation is the empirical study of health assets involving physical, mental, social, and spiritual dimensions, and is associated with well-being, depression, and health management competency. This study shows that higher HAV is associated positively with subjective well-being and negatively with depression. The findings that health assets might foster better quality of life and better mental health are consistent with the findings of earlier studies^9,15–17^. Systematic reviews by 23 publications from more than 13 different countries provided strong evidence that better self-rated health, psychological well-being and life satisfaction were associated with better health in older age^4^. Specially, the association of HAV with subjective well-being has provided an understanding of health assets with a focus on social, economic, and environmental predictors of well-being^4^.

Health management strategies and habits could be critical potential mediators between health assets and health^16^. This study shows that higher health management strategies are associated with higher HAV, and better health habits are associated with higher HAV over and beyond effects of demographic risk factors. These meaningful relationships of HAV with the health management strategy and habits suggest its potential as an instrument to measure the accumulated financial value of health assets^4,16^. This indicates that a focus on capability outcomes of health, rather than with health status, would alter the relative importance of preventing and treating different conditions^18^. Implementing an asset-based approach could uncover the health management strategy and habits of the individual and the community to overcome the challenges faced by global ageing^4^.

Health asset valuation can help understand how to improve health and well-being and maximize health assets^15^. This health asset valuation includes physical, mental, social, and spiritual health assets like physical strength, overcoming stress, social relationship, and volunteering^16,17^.

The influence accumulated from health determinants such as genes, health behaviors, medical care, and socioenvironmental factors during the lifetime produce the current health assets of an individual^19^. Socioeconomic circumstances are major determinants of people’s different health assets^2,19^, and financial resources consistently prove to be key economic health assets for an individual^4^. The lifelong HAV measure can capture the cumulative effect of the financial consequences of health over a long period of time^5^.

The economic value of losing people’s health could provide evidence of the benefits of good health to the nation economy and show the public the investment required for the infrastructure of a healthy society^2^. HAV also helps to tackle health inequities^20^. HAV could be used in the evaluation of strategies and policies aiming to create health assets and reduce health inequality^21^.

Given the increasing global importance for health, HAV model can be incorporated as a more challenging way of diagnosing individual or national capacity to improve health, developing multidisciplinary intervention to tackle health inequities, and evaluating the value for asset-based approach^16,20,21^. Rebalancing between the assets and deficit models by calculating max and loss of health asset value could help in better understanding the factors that influence health andthe actions needed to promote health^21–23^. Therefore, the HAV is a relevant strategy for enhancing health assets and motivating good health^16,20,22^. For example, it may provide some evidence to evaluate health policies and formulate national strategies against health crisis such as coronavirus disease 2019. However, a theoretical basis should be developed to provide evidence of the short- and long-term benefits of health to the individual, society, and nation^2,21^.

DespiteWHO’s definition of health, most approaches to health have focused still on the absence of illness^16^. This study shows that HAV based on the WHO’s definition of health, appears to be suitable for assessing health assets and has some possible policy implications^7^. Poor health is associated with not only the risk of lost earnings, but also the loss of valuable chances such as work-life balance, well-being, social activity, and various creative activities^5^. The answer to the question of the economic value of losing people’s health with a focus on physical, mental, social, and spiritual aspect of health will provide value of good health to the individual, nation and economy, and comprehensive evidence of economic consequences of physical, mental, social, and spiritual aspect of health accumulated over a long period of time at the individual and national level^4,5^. As the standard measure of the HAV created through the promotion of health in a country during a certain period^16^, HAV for each country can be calculated and compared using health state and GDP^24^. In addition, HAV can be useful in assessing the impact of health policies at the national level^7^. At the national level, as the health asset valuation can show long-term benefits of ‘salutogenic’ health for several years using the perceived health status reported annually by Statistics Korea or OECD, the HAV-based approach might be the single coherent model for evaluating the effectiveness of health policies to national health asset development^2,21^.

Whereas most approaches to health largely focus on the absence of illness, we investigated perceptions of health assets and their valuations by the general population based on the theory of salutogenesis and the idea of positive health^1^. These findings highlight the various possibilities of personalized health management strategies based on valuations of health assets at the individual. Health asset valuations may empower health management through various effective health programs and can also help to measure the effectiveness of these programs. Thus, health asset valuation can be a new evaluative tool based on salutogenic indicators^21^. Given the importance of health in daily life, health assets and their valuations can help in understanding the mechanisms by which health programs and policies promote health and tackle health inequalities^20^. These approaches can provide evidence-based estimates for short-term and long-term benefits of health investments at the individual, social, and national levels^2,20,21^. However, our findings suggest that more research on theoretical and evaluative issues related to health asset valuations is needed to convince individuals of their necessity for promoting health and tackling health inequalities^1,20,25^.

Although our results estimating the monetary value of health assets appear to have some meaningful findings, we need to acknowledge a few limitations. First, this study was cross-sectional, and the data set was an inevitable limitation of research because time series data did not become available. Second, in the process of converting the Likert scales used to measure the multidimensional health status into scores, the equal intervals were not uniform between the scales. The difference between ‘Excellent’, and ‘Very good’, is not the same as the difference between ‘Poor’, and ‘Very poor’. Third, in the monetary value of health, we weighed the four-health status so that the health status is anchored on a 0–1 for ‘worst’ to ‘excellent health’ to ‘full health scale’ based on the stepwise linear regression analysis of sum of four health dimensions that were designed to capture comprehensive health. The interpretation of HAV should be validated by future research. Four, although a representative group was sampled, the study was limited to Korea, and the extent to which these findings are generalizable to the global population is unclear. Collecting evidence of the effectiveness of asset-based approaches on health and well-being outcomes requires a change in individual and national attitudes, values, and practice^23^.

In conclusion, the HAV approach offers a new monetary value of health combining health with wealth. The findings of our study could be useful as a first estimate of value for health assets that can be used in making individual or political decisions of improving health or reducing health inequity.

The HAV approach offers a new monetary value of health that can be used in making individual or political decisions of improving health or reducing health inequity.

## Data Availability

The datasets used to analyze for the current study are available from the corresponding author on reasonable request.
